# The Influence of Incinerated Sewage Sludge as an Aggregate on the Selected Properties of Cement Mortars

**DOI:** 10.3390/ma14195846

**Published:** 2021-10-06

**Authors:** Teresa Rucińska, Anna Głowacka, Robert Sidełko

**Affiliations:** 1 Department of Building Physics and Building Materials, Faculty of Civil and Environmental Engineering, West Pomeranian University of Technology in Szczecin, al. Piastów 50a, 70-311 Szczecin, Poland; 2 Department of Environmental Engineering, Faculty of Civil and Environmental Engineering, West Pomeranian University of Technology in Szczecin, al. Piastów 50a, 70-311 Szczecin, Poland; 3 Faculty of Civil Engineering, Environmental and Geodetic Sciences, Koszalin University of Technology Poland, Śniadeckich St. 2, 75-453 Koszalin, Poland; robert.sidelko@tu.koszalin.pl

**Keywords:** cement mortar, recycling, sewage sludge

## Abstract

In line with the trend of using waste raw materials in the technology of building materials, experimental studies of cement mortars containing various amounts of fine-grained waste aggregate were carried out. The waste aggregate was based on an incinerated municipal sewage sludge which was mechanically crushed to an appropriate grading. Chemical and physical properties of the waste aggregate are presented. Mortars with varying amounts of waste aggregate as a replacement for natural sand were prepared. Study determines compressive strength and flexural strength up to 56 days. Properties such as capillary action, air content and thermal conductivity were determined. The results of the tests has shown that the incinerated waste sludge can be used as a partial or total replacement for natural aggregate. In mortars with waste aggregate, a favorable relation between flexural and compressive strengths was observed, which translates into increased strength of the interfacial transition zone. A significant increase in water absorption was observed for mortars containing high amounts of waste aggregate, which is directly related to its porous structure. Conducted studied prove that the aggregate obtained from incineration of the municipal sewage sludge can a feasible alternative for natural aggregates in production of masonry and rendering mortars for construction purposes.

## 1. Introduction

In light of the increasing demands for environmental protection [1,2,3] and, above all, restoring water quality, a growing number of newly build and modernized wastewater treatment plants is being observed. Organizing wastewater management in Poland has led to the development of not only the systems of wastewater collection and treatment, but also to the expansion of water supply and sewerage system.

Increase in the required wastewater treatment capacity is directly correlated with the amount of by-products in the wastewater. To those obtained during the treatment processes we can include, screenings, sand, fats, and initial and secondary sludges, which are removed in the largest amount.

Technology of sewage sludge management starts with drying sewage sludge in low temperature band dryers, to the content of minimum 90% d.m. (dry mass), and then incinerating it in stoker-fired boiler with moveable grate. While the ash from the incineration collected in the lower part of the boiler is removed periodically, the exhaust gases are directed to the secondary combustion chamber (temperature above 850 °C, the retention time—2 s), then to the heat exchanger, and last but not least are purified by filters.

The development of a technology that will reduce the amount of incinerated municipal sewage sludge, similarly like in a case of construction and demolition wastes [4,5,6,7], is desirable. Simultaneously, attention should be given to reducing raw materials usage in technological processes, as well as pollutant emissions during waste disposal and recycling [8,9,10]. The positive effects of such actions is favorable in a global scale.

Majority of studies focusing on the utilization of incinerated municipal sewage sludge (SS) in building materials indicate the usefulness of SS in the form of ash (SSA) in cement production or as a replacement in cement composites. Chin et al. [11] investigated the effects of incineration temperature (600 °C and 800 °C) and the influence of 10% cement replacement on the strength of cement mortar. They found that the raw sewage sludge mainly contains oxides of silicon, phosphorus, and iron (SiO_2_, P_2_O_5_, Fe_2_O_3_). After incineration, SSA ash mainly contains SiO_2_, Al_2_O_3_, Fe_2_O_3_, P_2_O_5_. Replacing 10% of cement with SSA retards the hydration process and early mortar strength, regardless of the SS incineration temperature. However, for SSA obtained at a incineration temperature of 800 °C, replacement of cement in the mortar improved slightly the strength after 28 and 90 days.

Chang et al. [12] also investigated the possibility of reusing SSA in cement composites. They used the SSA from the incineration of municipal sewage sludge at 800 °C. The SSA composition showed a low content of heavy metals, including Pb, Cd, Cr, and Cu. The SSA contained mainly SiO₂, CaO, Fe₂O₃, and MgO. The study has shown that SSA is a porous material with an irregular surface that caused an increased water absorption, resulting in a decrease of the workability. Use of the SSA decreased the compressive strength of cement composites. However, it was found that the addition of 10% SSA as a cement replacement did not significantly affect the properties of cementitious materials. The physical, chemical, and mineralogical characteristics of SSA and evaluation of its application in cement-based materials were also described by Cyr et al. [13] and Pan et al. [14].

Garcés et al. [15] tested the application of SSA in CEM I (Portland cement) and CEM II (Portland composite cement) cements with different clinker contents. Tests were performed for SSA replacement of 10% to 30% by weight of cement. Best results were obtained for the CEM II/B-M (V-LL) 42.5R (Portland composite cement, class 42.5 with high early strength, identified as R—in accordance with EN 197-1) with 10% replacement rate. Krejcirikova et al. [16] determining the chemical parameters and granulometry of the SSA and analyzed the selected physical properties of the cement-ash mortar. It was observed that the particles of SSA were larger than those of cement. The presence of SSA in the mortar, increased the porosity of the structure, which resulted in a decrease in the 28-day compressive strength. It was also observed that the SSA increased water absorption, but on the other hand did not affect the sorption isotherms of the studied mortars.

The influence of incinerated sewage sludge on the properties of cement during clinker burning was studied by Lin et al. [17]. Authors analyzed the effect of 0.5–15% addition of the SSA to clinker. The mix was then burned at 1450 °C for 2 h. This process yielded a very similar composition to Portland cement. It was observed that the amount of the C_2_S phase increased with the increase of the SSA content. Similarly, the setting time increased with the content of the SSA. The compressive strength of studied mortars decreased with increasing amounts of the SSA.

As shown, the use of incinerated municipal sewage sludge is limited to the application of SSA as an additive in cement production or as a cement substitute in cement composites. The use of SS as fine aggregate in the technology of mortars or cement concretes has not been reported, hence the authors’ interest in this topic. This study determines the basic parameters of cement mortars with fine aggregate obtained from the process of incineration of municipal sewage sludge. Use of the SS on an industrial scale can contribute to the reduction of environmental pollution and use of natural aggregates. This combines the idea of sustainable development with the ever-increasing necessity of secondary processing and near-zero waste management (virtually waste-free).

## 2. Materials and Methods

### 2.1. Materials

**Figure 1:**
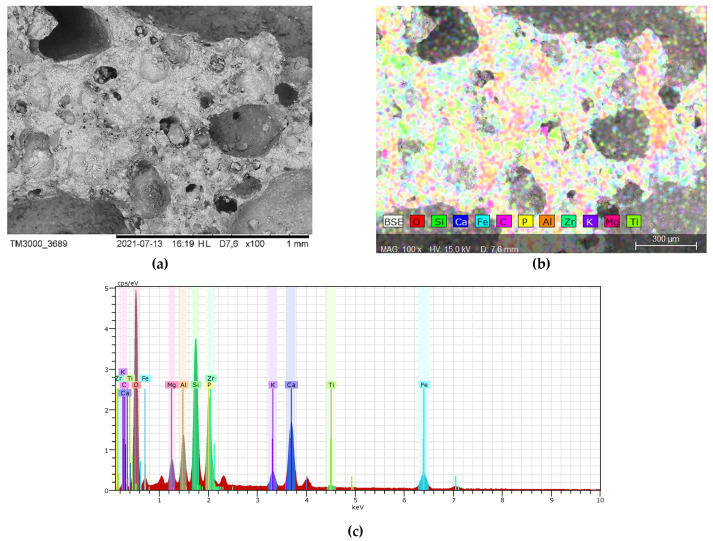
(**a**) Micrograph of the SS surface; (**b**) distribution of identified elements; (**c**) elements found in the SS.

Preliminary tests of the slag produced by incineration of sewage sludge were conducted as presented in [18]. The micrograph and element identification by means of EDS are presented in Figure 1.

The grading of the SS showed that 48.4% are fractions between 0.063 and 1.25 mm. The results has shown that the waste in accordance to Polish waste database can be categorized as bottom ash and slag. This means that it can be used as an alternative source of phosphorus for direct application to soil treatment, the production of fertilizers, and organic-mineral and as a recycling construction aggregate for the production of mortars and concretes [19,20,21].

**Figure 2:**
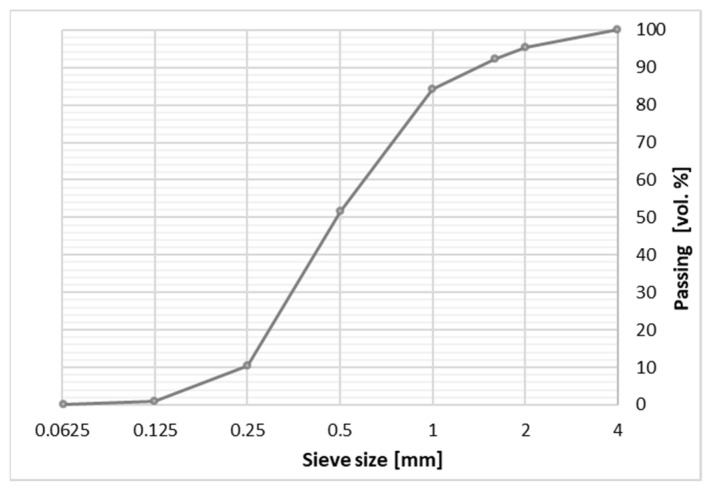
Grading of the natural aggregate.

The determination of slag and natural aggregate grading is presented in Figure 2. The waste aggregate (<2 mm) was composed of grains formed during the combustion of SS and obtained by mechanical crushing of larger SS particles.

**Figure 3:**
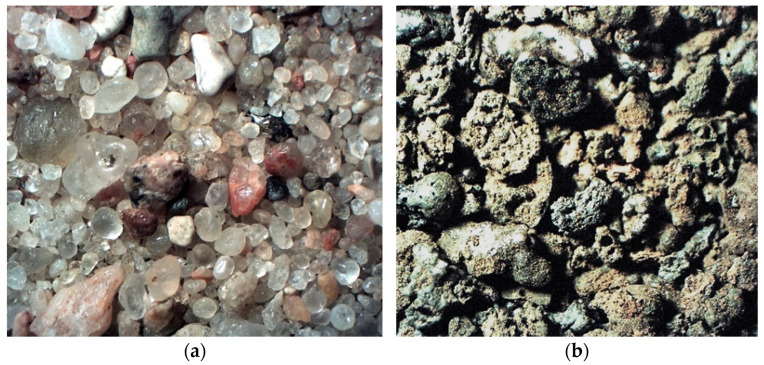
(**a**) Natural aggregate. (**b**) Incinerated sewage sludge aggregate (magnification of 10×).

Fine aggregate (<2 mm) used in the study is presented in Figure 3a. The aggregate was obtained from a Bielinek aggregate mine (Bielinek, Poland). The aggregate meets the requirements of the EN 12620. The aggregate was oven-dried at 70 °C for 20 h to eliminate residual moisture.

**Figure 4:**
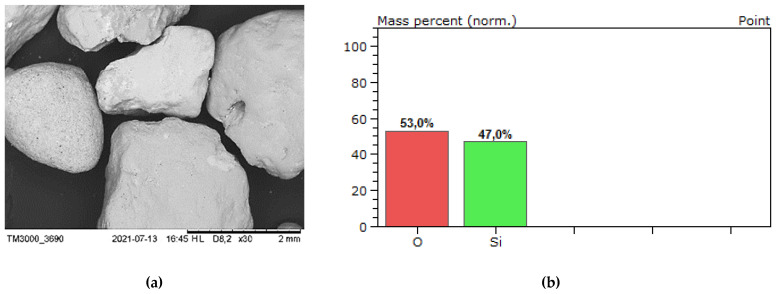
(**a**) Natural aggregate (A_NS_) grains—magnification of 30×; (**b**) identification of the elements.

**Figure 5:**
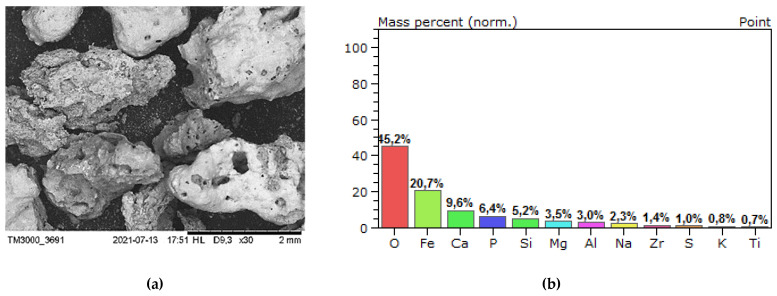
(**a**) Waste aggregate (A_FSS_) grains—magnification of 30×; (**b**) identification of elements.

The ash-slag generated from thermal disposal of municipal sewage sludge (Figure 4), comes from the Pomorzany Waste Water Treatment Plant (Szczecin, Poland). It was produced by drying the sludge in a contact dryer and then incinerating it in a moving grate boiler [21]. The material is porous with open-pores (Figure 5a), therefore has low mechanical strength and increased water absorption, which can be observed during mixing of the mortar [3].

The waste aggregate with grains up to 2 mm (A_FSS_) was designed to correspond to the grading of the natural sand (Figure 2). Dust and smaller particles were obtained by brushing in a ball mill. The aggregate was oven-dried at 70 °C to constant mass.

The EDS analysis (SEM—Hitachi Tabletop Microscope TM3000, Tokyo, Japan, EDS—QUANTAX 70, Bruker Nano GmbH, Berlin, Germany) was performed to identify the chemical elements in both aggregates (Figure 4 and Figure 5). As shown in Figure 5b, no heavy metals has been identified in the ash-slag or their concentration was below the detection level. However, the occurrence of phosphorus is problematic. It is known that phosphates (P_2_O_5_) content affects the cement setting [22,23].

Phosphates in the presence of lime (Ca) found in the fresh paste can cause precipitation of the poorly soluble calcium phosphate Ca_3_(PO_4_)_2_ on the surface of cement grains. The compound forms a fine crystalline and poorly permeable layer, which limits the hydration process of the cement. Furthermore, poorly soluble phosphates can crystallize in the pores of the paste. This results in an increase in setting time and a decrease in strength, especially early age. Therefore, their presence is problematic, especially when the composition of municipal sewage sludge is not constant and the amount of phosphorus changes.

Due to their porous nature, use of waste aggregates requires a special procedure to regulate the amount of water dosed into the mix. The total water must incorporate not only batch water but also additional water, reflecting the water absorption of the aggregate. The water absorbed by grains released during the maturation of concrete causes self-curing improving the strength of interfacial transition zone (ITZ). Improvement in the relation of the tensile strength and compressive strength was also observed. It is caused by irregular shape of the grains of the A_FSS_ and rough surface [24]. Excessive water, that was not consumed by the hydration, evaporates during maturation of concrete, leaving the micropores, which weaken the structure of the composite.

### 2.2. Mix Preparation

Six cement mortars with different amount of waste aggregate obtained from the incineration of municipal sewage sludge (A_FSS_) were prepared. The reference mortar (MR) contained only natural aggregate (A_NS_), while the other five differed in the percentage of waste aggregate (MSS10, MSS20, MSS30, MSS50, and MSS100, where 10, 20, 30, 50 and 100 is the percentage of waste aggregate in the total volume of fine aggregate in the mortar). Investigations included determination of compressive and flexural strengths (up to 56 days) for mortars with varying amount of recycled aggregate.

Use of waste aggregates is in line with the Regulation No. 305/2011 of the European Parliament and European Council of 9 March 2011, which encourages introduction of construction products with recycled and raw materials.

For the purpose of the study, Portland cement CEM I 42.5R manufactured by GÓRAŻDŻE CEMENT (Górażdże, Poland) was used. This cement is characterized by high early (2 days) and standard (28 days) compressive strength, rapid strength development, stable quality, and high hydration heat.

Mixes were prepared using tap water, which meets standard requirements. For the purpose of this study, a standard mortar was used as a reference (Water/cement (W/C) = 0.5 W/C = 0.5). Mixes with waste aggregate were modified with a MasterGlenium ACE 430 (BASF) plasticizer in the amount of 1.5% of cement mass. To obtain the assumed slump flow of <140 mm for mixes with waste aggregate, additional water was added in the amount presented in Table 1.

The cement to aggregate ratio of 1:3 was assumed in the study. The water-cement ratio of 0.5 was initially assumed. The composition of the mixes corresponds to a standard cement mortar found in EN-196-1 which was adopted as a reference mix. The proportion of 1:3 refers to the mass of cement and fine aggregate. Waste aggregate (A_FSS_) was used as a replacement of the natural sand (A_NS_) by 10%, 20% and 30%, 50%, and 100% of volume, due to differences in density. Use of increased amount of the A_FSS_ resulted in an increase in added water to the mix.

The notation and composition of prepared mixes are as follows:

MR—reference mix, 100% A_NS_

MSS10—mix with 10% A_FSS_ + 90% A_NS_

MSS20—mix with 20% A_FSS_ + 80% A_NS_

MSS30—mix with 30% A_FSS_ + 70% A_NS_

MSS50—mix with 50% A_FSS_ + 50% A_NS_

MSS100—mix with 100% A_FSS_ + 0% A_NS_

Density of particular mix components is presented in Table 2.

**Figure 6:**
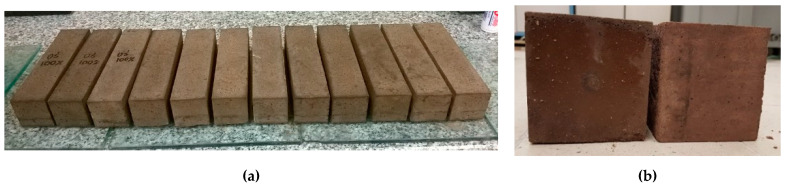
Specimen for determination of hardened properties; (**a**) prisms 40 mm × 40 mm × 160 mm; (**b**) samples 100 mm × 100 mm × 50 mm.

The components of the mix were mixed together and the determination of consistency (EN 1015-3) and air content (EN 1015-7) was performed. The consistency was the key characteristic. As the waste aggregate A_FSS_ is highly porous (Figure 5a), to achieve the same consistency water was added to the mix. This means that except the initially assumed batch water, additional water that was absorbed by the aggregate was added. The specimen (40 mm × 40 mm × 160 mm) were prepared for the flexural and compressive strength tests (EN 1015-11) as well as dry bulk density (EN 1015-10), water absorption coefficient due to capillary action (EN 1015-18) and water absorption (Figure 6).

Due to the requirements of the measuring apparatus, specimens with dimensions of 100 mm × 100 mm × 50 mm were prepared for the determination of thermal conductivity coefficient (Figure 6). The specimens were stored in a chamber with RH > 95% and T = 20 °C until tested. Protocols for the determination of non-stationary thermal conductivity coefficient found in ISOMET 2104 apparatus were used. The measurement was performed using contact sensors with a fixed measuring range.

The samples were oven-dried at 70 ± 5 °C. The flexural and compressive strengths were tested in a Walter+Bai AG strength testing machine (Walter+Bai AG Testing Machines, Löhningen, Switzerland). The air content of fresh mortar was determined using the pressure method, while the water absorption was determined using the soaking method. The consistency of fresh mortar was determined using a slump flow table.

## 3. Results and Discussion

The results of tests of mortars with waste aggregate were compared to the reference mortar. This allowed to indicate the effect of increasing the amounts of waste aggregate on mortar properties.

### 3.1. Fresh Mortar Consistency

**Figure 7:**
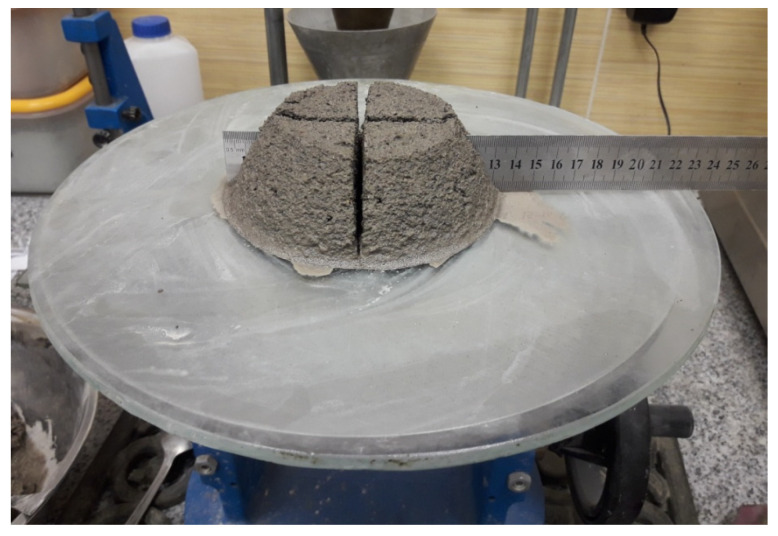
Slump flow determination.

All the tested mortars have a slump flow of <140 mm (dry consistency) determined in accordance with EN 1015-3. However, when determining the consistency of fresh mortar with at least 20% A_FSS_ bleeding was observed (Figure 7). One of the reasons may be due to the difference in density between the water and the rest of the mortar components—the components displaced water from the paste during settlement. A second reason is that the waste aggregate used in these mortars had a high absorption rate, requiring additional water to be added to the mix. It is concluded that when the sample got vibrations from the table during the test, the absorbed water flowed out of the pores of the aggregate grains, resulting in leakage at the base of the sample.

Table 3 presents the mean values of fresh mortar slump test. The diameter was measured in two perpendicular directions. The values were rounded up to 1 mm.

### 3.2. Air Content in Fresh Mortar

**Figure 8:**
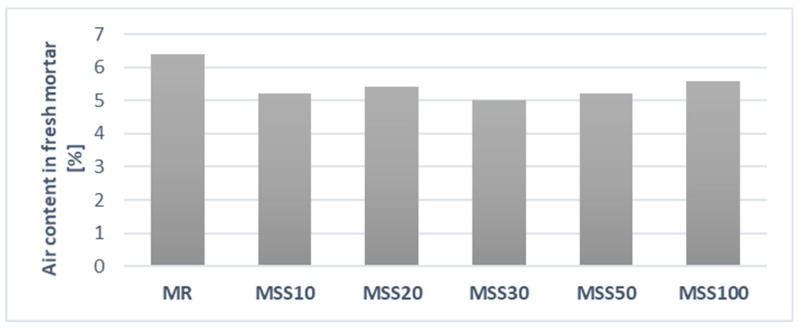
Air content in fresh mortar.

Results of the air content determination of fresh mortar is presented in Figure 8. The reference mortar showed the highest air content. On the other hand, mortars with varying amounts of waste aggregate have similar air content. This may be due to the fact that the MSS20–MSS100 mixes required additional water, which resulted in a more favorable workability of the mix compared to the reference mortar. The MSS10 mortar, despite having only 10% of waste aggregate and W/C = 0.5 had equally lower air content as the MSS20–MSS50 mortars. This means that the MSS10 mix had better workability than the reference mortar.

### 3.3. Sample Molding

During the molding of the test specimens, it was observed that the addition of 20% to 30% of slag delayed the final setting time by 48 h, while 50% and 100% delayed it by up to 72 h. Only after this time was it possible to demold the samples without damaging them. Slowing down the setting of the binder in mortars with burnt municipal sewage sludge may result not only from the presence of phosphates (P_2_O_5_). To obtain a required consistency of fresh mortar it was necessary to add an increased amount of water. The so-called “extra” water absorbed by the grains of waste aggregate does not take part in the hydration process, which certainly contributes to the delay in setting time.

### 3.4. Dry-Hardened Density

**Figure 9:**
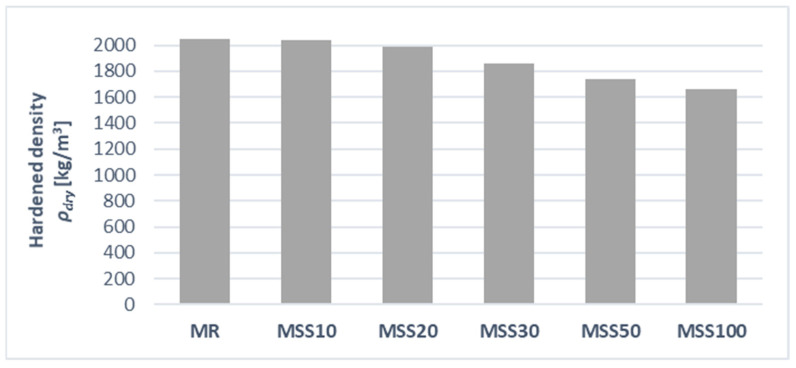
Hardened density of mortars.

The hardened density of the mortars clearly decreases with an increase in the amount of waste aggregate, which is primarily related to its lower bulk density—Figure 9. The reference mortar achieved a hardened density of 2050 kg/m^3^, while hardened density of the MSS10 was 2040 kg/m^3^. A more significant difference in density is seen when the amount of waste aggregate is 30% and above.

### 3.5. Mortar Strength

**Figure 10:**
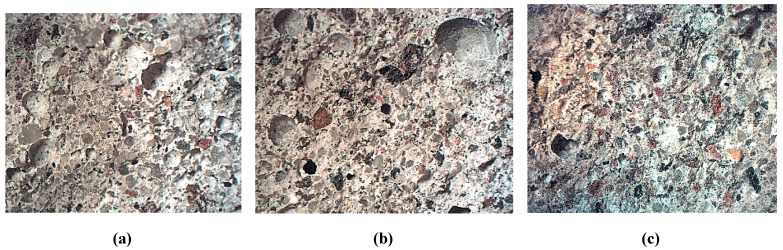
Cross-section of mortars—magnification of 10×, (**a**) 10% of A_FSS_; (**b**) 20% of A_FSS_; (**c**) 30% of A_FSS_.

Flexural and compressive strengths were determined after 7, 28, and 56 days of maturation. Figure 10 shows the examples of cross-section images obtained after flexural strength testing. It can be seen that the waste aggregate grains are uniformly distributed, with clearly visible broken A_FSS_ grains indicating that they are the weakest parts of the structure.

**Figure 11:**
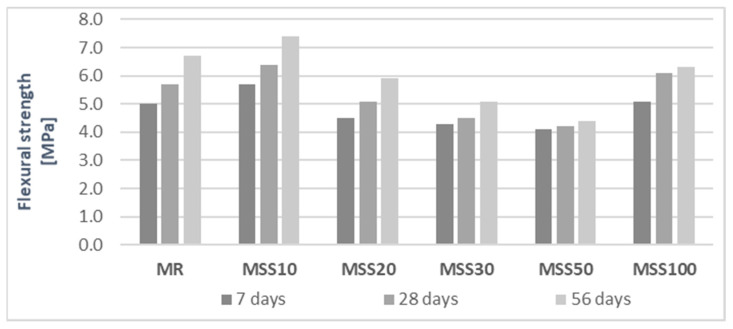
Flexural strength of mortars after 7, 28, and 56 of maturation.

The result of flexural strength determination (Figure 11) has shown that the addition of 10% A_FSS_ significantly improved this parameter. Increasing the content of A_FSS_ up to 50% decreased the flexural strength of the mortars. The MSS100 mortar, with only the waste aggregates had higher flexural strength than mortar with mixed aggregate content. The grains of these aggregates have different structure, porosity, surface roughness, and shape. It can be concluded that not only the porous structure of the A_FSS_ aggregate affects the flexural strength, but also the arrangement of both aggregates withing the mortars. The results of the flexural strength of the MSS100 mortar prove that the rough surface of the grains improves the interfacial transition zone (ITZ). Increased surface are due to high open-pores, which allows for a penetration of the paste. Increased number of pores improves the effect of internal curing. It was observed that the flexural strength of the MSS100 mortar compared to its compressive strength is more affected by the porous structure [24].

**Figure 12:**
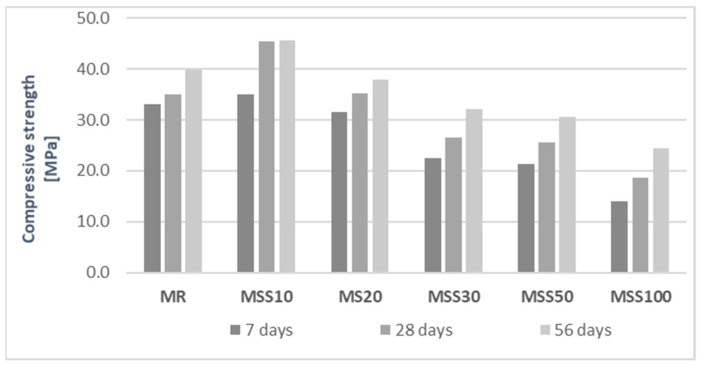
Compressive strength of mortars after 7, 28, and 56 days of maturation.

Considering the compressive strength (Figure 12) in accordance with EN 998-1 mortars with up to 50% of waste aggregate could be classified as CS IV (compressive strength at 28 days > 6 N/mm^2^), allowing them to be used in concrete floors, masonry, or rendering. In accordance with EN 988-2 the mortars are considered as Md (compressive strength at 28 days > 25 N/mm^2^). This means that their compressive strength exceeded 25 MPa. The mortar MSS100 with only waste aggregate had lower strength allowing to categorize it as masonry mortar M15 (compressive strength at 28 days ≥ 15 N/mm^2^) and CS IV for rendering.

Analyzing the flexural and compressive strength values of individual mortars, it can be concluded that an increase in the amount of waste aggregate decreased the brittleness of mortars. Mortars with A_FSS_ aggregate, which is highly porous and has a rough surface (Figure 5), exhibit better cooperation at the interfacial transition zone [24]. The ratio of flexural strength to compressive strength of the reference mortar is around 16%, while in case of mortar with 100% of waste aggregate this ration increased to approximately 32%.

### 3.6. Thermal Conductivity Coefficient

**Figure 13:**
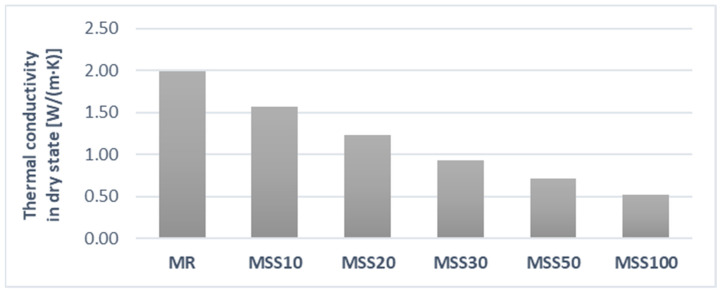
Thermal conductivity in dry state of studied mortars.

Thermal conductivity was another characteristic determined in the study (Figure 13).

**Figure 14:**
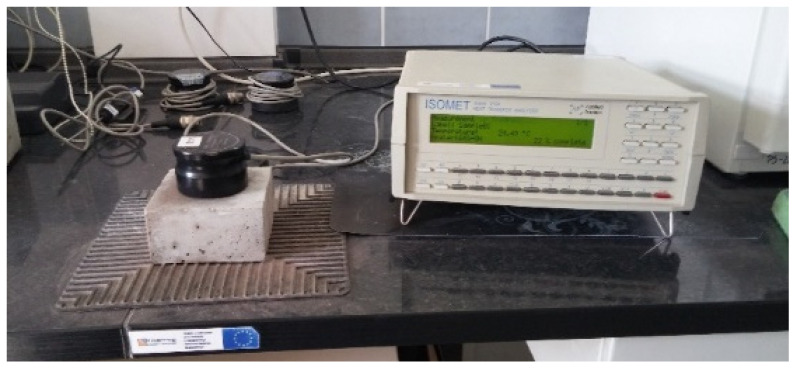
Thermal conductivity coefficient determined in a non-stationary method.

As can be seen in Figure 9 and Figure 13, obvious correlation between bulk density and thermal conductivity is visible. The tests were conducted using the non-stationary method with the ISOMET 2104 apparatus (Applied Precision Ltd., Bratislava, Slovakia) (Figure 14).

Results show that increase of the waste aggregate content in the composition of mortars increases the thermal insulation properties, which in the case of masonry mortars translates directly into a reduction in the intensity of thermal bridges at the mortar-masonry interface. This is most evident with 100% waste aggregate in the mortar composition.

### 3.7. Water Absorpion Due to Capillary Action

**Figure 15:**
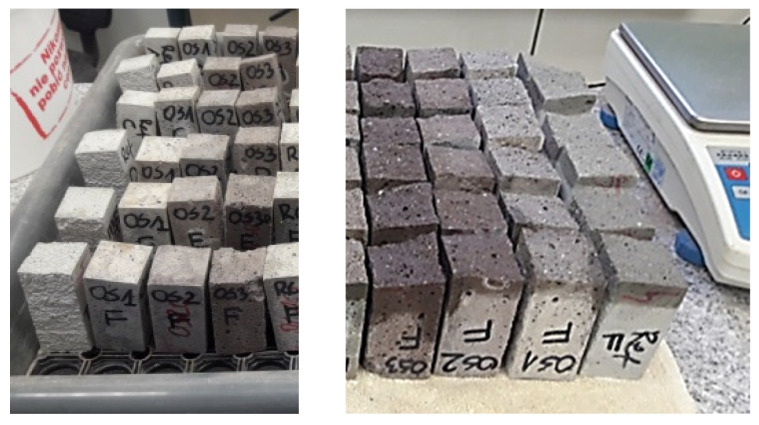
Specimen during the determination of water absorption coefficient due to capillary action.

**Figure 16:**
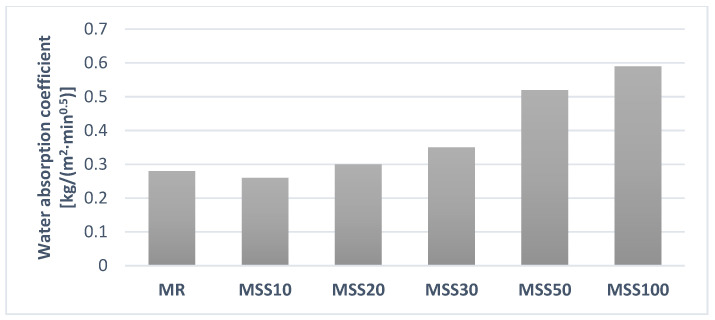
Water absorption coefficient due to capillary action of hardened mortar.

According to EN 998-1 for rendering mortars, MR, MSS10, MSS20, and MSS30 were classified as W1 (the mean coefficient of water absorption of the sample of mortar due to capillary action C_m_ ≤ 0.4 kg/m^2^·min^0.5^) with regard to the obtained value of the water ab-sorption due to capillary action (Figure 15). Mortars MSS50 and MSS100 mortars were classified as W0 (Figure 16).

Such a high value of the water absorption coefficient is a result of the porous structure of the waste aggregate with high prevalence of fine pores (Figure 5a). The higher the amount of the waste aggregate the higher the values of this coefficient. However, dynamic increase in the value is visible for mortars with at least 30% of waste aggregate. Results of this test show limited application of those mortars in areas exposed to water.

Capillary action is caused by capillary forces. The magnitude of these forces is related to the rate of capillary action and this depends on the size of the pores and their structure. The increase in capillary action correlated with the increase of number of pores in the structure, and its dynamics depends on the pore size [25,26].

### 3.8. Water Absorption

**Figure 17:**
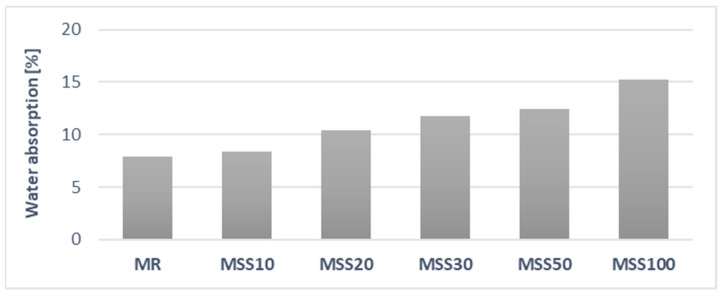
Water absorption of studied mortars.

Water absorption was determined through soaking (Figure 17).

The mass measurement in the saturated state clearly indicate an increase in water absorption as the amount of waste aggregate A_FSS_ in the mortar composition increases. Water absorption is directly correlated to the porosity of the aggregate. It is worth noting, however, that at 10% of A_FSS_, the increase in absorption was not as significant as in other cases.

## 4. Conclusion

The aim of this study was to determine the feasibility of using a waste fine aggregate acquired by incineration of municipal sewage sludge for production of mortars for plastering and rendering. Based on the acquired results of tests that are referred by the categories of mortars found in EN 998-1 and EN 998-2 the following conclusions can be drawn:

1. Waste aggregate obtained from incineration of municipal sewage sludge, can be a valuable component of mortars. All analyzed mortars showed suitability for construction purposes.
2. The porous structure and open pores of the waste aggregate results in significant absorption of batch water during mortar mixing, which requires adding additional water experimentally to obtain the desired consistency. This was not observed for 10% of aggregate replacement.
3. In mortars with waste aggregate, a favorable relation between flexural and compressive strengths was observed, which translates into increased strength of the interfacial transition zone.
4. The strength of the mortars allows them to be categorize in accordance with EN 998-1 and EN-998-2. As the proportion of waste aggregate increases, the compressive strength of MSS20-MSS100 mortars decreases rapidly.
5. Water absorption coefficient of mortars increases with the increase of the porous waste aggregate.
6. A significant increase in water absorption was observed for mortars containing high amounts of waste aggregate, which is directly related to its porous structure.

Conducted study proves that the aggregate obtained from incineration of the municipal sewage sludge can be a feasible alternative for natural aggregates in production of masonry and rendering mortars for construction purposes.

## Figures and Tables

**Table 1:** Total water amount in prepared mixes.

Mix	MR	MSS10	MSS20	MSS30	MSS50	MSS100
Total W/C Batch water + added water	0.5	0.5	0.65	0.72	0.95	1.16

**Table 2:** Density of mix components.

$\mathrm{Density}\;[\frac{g}{cm^{3}}]$				
Natural Sand 0–2 mm	Waste Aggregate 0–2 mm	CEM I 42.5 R	Water	MasterGlenium ACE 430
2.65	2.86	3.11	1.0	1.06

**Table 3:** Mean values in slump test in accordance with EN 1015-3.

Mix	MR	MSS10	MSS20	MSS30	MSS50	MSS100
Mean slump [mm]	135	133	133	133	134	133

## Data Availability

The data presented in this study are available upon reasonable request from the corresponding author.
